# Apparatus design and behavioural testing protocol for the evaluation of spatial working memory in mice through the spontaneous alternation T-maze

**DOI:** 10.1038/s41598-021-00402-7

**Published:** 2021-10-27

**Authors:** Raffaele d’Isa, Giancarlo Comi, Letizia Leocani

**Affiliations:** 1 Experimental Neurophysiology Unit, Institute of Experimental Neurology (INSPE), San Raffaele Scientific Institute, IRCCS-San Raffaele Hospital, Milan, Italy; 2 Vita-Salute San Raffaele University, Milan, Italy; 3 Casa di Cura del Policlinico, Milan, Italy

**Keywords:** Working memory, Learning and memory, Spatial memory, Neuroscience

## Abstract

Spatial working memory can be assessed in mice through the spontaneous alternation T-maze test. The T-maze is a T-shaped apparatus featuring a stem (start arm) and two lateral goal arms (left and right arms). The procedure is based on the natural tendency of rodents to prefer exploring a novel arm over a familiar one, which induces them to alternate the choice of the goal arm across repeated trials. During the task, in order to successfully alternate choices across trials, an animal has to remember which arm had been visited in the previous trial, which makes spontaneous alternation T-maze an optimal test for spatial working memory. As this test relies on a spontaneous behaviour and does not require rewards, punishments or pre-training, it represents a particularly useful tool for cognitive evaluation, both time-saving and animal-friendly. We describe here in detail the apparatus and the protocol, providing representative results on wild-type healthy mice.

## Introduction

Memory can be broadly defined as the persistence of previously acquired information^1^. Importantly, memory is not a unique faculty but can be subdivided into different subsystems. On the basis of the duration of its retention, we can distinguish between short-term memory (STM), which ranges from seconds to minutes, and long-term memory (LTM), which is characterized by a duration of days, years or even decades. The expression “working memory” was first coined by George Armitage Miller (1920–2012), Eugene Galanter (1924–2016) and Karl Pribram (1919–2015) in their seminal book *Plans and the Structure of Behavior* in reference to a memory store for action plans^2^. The concept of working memory partially overlaps the one of STM and although many authors subsequently utilized the expressions as synonyms, working memory actually refers more specifically to a cognitive buffer that stores information and permits its manipulation to guide decision-making and behaviour^3^. Its capacity is limited, as well as its duration, and its usefulness is strictly related to the execution of a specific task. On the other hand, on the basis of its content, memory can likewise be divided into several subtypes, including visual, auditory, tactile, olfactory, gustatory, interoceptive and spatial. In particular, spatial working memory designates temporary spatial information needed to carry out a specific behaviour.

Since the pioneering studies of Willard Stanton Small (1870–1943) at the end of the 1890s^4^ mazes have been widely employed in behavioural neuroscience to investigate experimentally memory processes through animal models, in particular rodents, such as mice and rats.

One of the most well-established tests of spatial working memory is the spontaneous alternation T-maze. The T-maze is a T-shaped apparatus allowing a choice between two opposite arms. A first T-maze apparatus was designed at the beginning of the 1910s by Robert Yerkes (1876–1956) at Harvard University for the study of invertebrate cognition, in particular learning processes in the earthworm^5^. In the 1920s, Edward Tolman (1886–1959) applied the T-maze to the study of rodent cognition and was the first to report the phenomenon of spontaneous alternation^6^. This phenomenon was better defined and investigated in the 1930s by Wayne Dennis (1905–1976)^7–9^, who also coined the expression “spontaneous alternation”^10^. Basically, spontaneous alternation T-maze is based on the natural tendency of rodents to prefer exploring a novel arm over a familiar one, which induces them to alternate the choice of the goal arm^6,10–13^. After a first choice in the sample trial, in the second trial the arm which has not been previously explored is preferred. In the third trial, when both arms have been visited, the arm with the highest degree of novelty (the arm explored longer ago) is preferred. Since this behaviour does not require pre-training and relies merely on the natural attraction of rodents for novelty, alternation has been defined as spontaneous. An alternative explanation for alternation is that mice have an innate tendency to shift. For example, visiting a place that contains a food reward leads to a win-shift behaviour, while visiting a place that does not contain food leads to a loose-shift behaviour. In both cases (innate novelty-seeking behaviour or innate shifting behaviour), during the task, in order to successfully alternate choices across trials, an animal has to remember which arm had been visited in the previous trial, which makes spontaneous alternation T-maze an optimal test for spatial working memory. Over the past century, spontaneous alternation has been found in a wide variety of mammalian species, including rats^6^, mice^14^, hamsters^15^, guinea pigs^16^, rabbits^17^, gerbils^18^, ferrets^19^, opossums^20^, marmosets^21^ and cats^22^, and also in non-mammals such as pill bugs^23^, garden woodlice^24^, marine crabs^25^, fruit flies^26,27^, goldfish^28^ and zebrafish^29^.

Regarding the murine procedure, in the sample trial mice are released in the stem of the T-maze (the start arm) and let free to choose one the two lateral goal arms to explore. After the entrance a guillotine door is shut down and the animal is confined in the chosen arm for a fixed amount of time (for example 30 s), at the end of which it is removed and newly placed in the start arm with all the doors raised for the test trial. A series of test trials is then carried out (usually from 5 to 12, depending on the protocol), with an intertrial interval (ITI) that can range from virtually zero to 20, 40 or even 60 s. Percentages of alternation across the test trials are calculated as an index of working memory. Healthy wild-type animals generally display a percentage of alternation around 70–75%, far above chance level (50%). The task is sensitive to the length of the delay period between trials, with spontaneous alternation rates that are inversely related to the ITI^30,31^. As Morris water maze^32^, another common tool for spatial memory assessment, also this test is hippocampus-dependent, but it is typically considered more sensitive in evaluating hippocampal function, as both tests detect complete lesions of the hippocampus but spontaneous alternation T-maze is better at detecting partial dysfunctions^13,33^.

Different versions of the T-maze have been developed, which can be distinguished on the basis of which option is selected for key features of the test. In particular:

- spontaneous versus rewarded alternation: alternatively to the spontaneous alternation version, there is a version in which correct alternations are systematically rewarded^34,35^. In this version, target arms (arms that were not explored in the previous trial) are baited with a food pellet. The alternation behaviour is not spontaneous, but reinforced by the experimenter. The spontaneous alternation version has several advantages compared with the rewarded version. First of all, since it relies on a natural behaviour and not a trained one, it has a higher ecological validity and it is not influenced by experimenter-imposed sources of motivation. Moreover, it does not require food deprivation and is hence less stressful for the animals. Finally, it is less time-consuming, since it can be performed in one single session, while the rewarded version requires several days before starting (a period of food restriction is necessary, along with habituation to eat the food used in the maze). Notably, the spontaneous alternation version also appears to be more sensitive than the rewarded alternation version in detecting cognitive impairments. Indeed, mice with lesion of mammillary bodies tested with an ITI of 30 s were impaired in spontaneous but not rewarded alternation. Increasing the difficulty of the task by augmenting the ITI (to 50 and 180 s) showed that for rewarded alternation the cognitive impairment appeared only with an ITI of 180^36^. Furthermore, septal lesions induced a reduction of T-maze performance that for rewarded alternation was transient and could be recovered during the sessions of testing, while for spontaneous alternation the deficit was persistent and unrecoverable^37^.
- discrete versus continuous trials: in the discrete trials version, each trial is temporally separated, as animals return to the start arm each time. On the other hand, in the continuous trials version (generally performed with a Y-maze, in which three equal 120° angles are formed between arms) animals are left in free exploration of the maze for a determined amount of time or number of arm visits. Every time an animal chooses to enter one the two arms that was not visited in the previous trial, a correct alternation is scored. Basically only re-entering the arm that has just been left is considered an error. An alternative method for scoring alternations in the continuous version is the one based on triplets. In this case, an alternation is scored when all three arms are explored consecutively. The first two arm visits can be considered samples. For all the following visits, an entrance is rated alternation if the chosen arm has not been visited in the two previous choices. So, for instance, if the three arms are named A, B and C, in the sequence B–A–C–A–B there would be two alternations. The continuous alternation task has the advantage of avoiding possible stress deriving from handling between test trials. But one great limit of the continuous version is that it may not be able to detect hippocampal dysfunctions. Since hippocampal lesions frequently lead to side preference (turning always left or always right), in the continuous version these animals would appear to have normal memory (with both methods of rating), while the discrete version would correctly show that they have a very low alternation rate (often significantly lower than chance level). Robet Gerlai developed a continuous alternation procedure in the T-maze that avoids this sensitivity defect of the continuous alternation Y-maze^38,39^. Nevertheless, compared with the discrete trials T-maze, both continuous versions have the limitation that time of confinement in the chosen arm is uncontrolled. Furthermore, the interval between trials is not fixed and cannot be controlled in the continuous versions. It is particularly important to use a fixed ITI, since spatial working memory is delay-dependent. Indeed, number of correct alternations progressively decreased with ITIs ranging from 0 to 600 s^40^. Additionally, in the discrete trials procedure, the ITI can be manipulated as experimental variable, in order to increase the difficulty of the task. In some cases, a cognitive impairment is absent with a low intertrial interval, but appears with higher intervals. For instance, social defeat stress led to impaired spontaneous alternation with 90 but not with 60 or 30 s of ITI^41^. Cognitive enhancement effects also may be delay-dependent. For example, modafinil enhanced alternation rates at long ITIs (60 and 180 s), but not at a short ITI (5 s)^42^.
- enclosed versus open arms: rodents tend to be less anxious and more prone to explore in mazes with enclosed arms. As demonstrated by the elevated plus maze, which features two open and two enclosed arms, mice strongly prefer enclosed arms, spending approximately 75% of the time in the enclosed arms and 25% in the open^43^. T-mazes with open arms, that generate anxiety in rodents, will often require one or more habituation sessions before starting, in order to allow the animals to familiarize themselves with the apparatus. Moreover, for a healthy mouse it is easy to escape from the apparatus by jumping off the open arms. For these reasons, enclosed T-mazes should be considered a better option for the study of spatial working memory.

The T-maze can also be employed to study habit learning. By rotating the maze 180°, the T-maze can indeed be used as a cross (or plus maze). The place/response cross maze task, originally conceived by Tolman in the 1940s^44,45^ and resumed by Mark Packard and James McGaugh in the mid 1990s^46,47^, is one of the most efficient tools to evaluate place versus response learning^48^. In this procedure the experimenter releases the animals in the inferior arm of a cross maze surrounded by specific environmental visual cues and trains them to find a food reward which is constantly placed in one defined lateral arm (for example the right one), while keeping closed the two remaining arms (left and superior). When the training is over, in the test session the superior arm is selected as new releasing point, if a cross maze is used, or the maze is rotated 180°, in case a T-maze is used. After T-maze rotation, all external visual cues surrounding the maze remain stationary, along with the position of the experimenter. The arms of the maze are all identical in appearance and after the rotation odour cues are accurately removed with an ethanol solution, so that no intra-maze cues are present and only extra-maze cues are available. Animals following a place strategy will utilize the extra-maze visual cues as orientation points and will execute a behavioural response that is the opposite of the one they used to perform in the training (hence a left turn this time), demonstrating goal-oriented behaviour. On the other hand, animals adopting a response strategy will execute the same right turn of the training, revealing a rigid and inflexible behaviour, which features a specific motor response based on a stimulus–response association (known as habit). While goal-directed navigation is environment-centered (allocentric), the latter strategy is body-centered (egocentric). The characteristics of the mazes can be determinant for the adoption of one strategy or the other. For instance, a T-maze with open arms or enclosed arms with transparent walls allows allocentric orientation, while in a maze with high obscured walls impeding the vision of distal cues and where no intra-maze cues are present the egocentric strategy will be promoted. Indeed, it has been shown that in environments with abundant extra-maze stimuli, the allocentric place strategy is preferred over the egocentric response strategy, while the opposite is true in environments with few or no extra-maze cues^48^.

Finally, T-mazes and Y-mazes can be utilized also to assess short-term habituation to a novel environment^49–56^. Basically, animals are exposed for a determined amount of time (for example 15 min) to a training session with one arm closed and two open. After a delay (which can range from 1 min to few hours) a novelty preference test is performed. In the test session, all three arms are open and free exploration of the maze is allowed. Healthy animals tend to prefer the novel arm over a familiar one and time spent in the novel arm is measured as an index of short-term spatial memory.

In the present paper we will focus on spontaneous alternation testing, carried out through discrete trials in an enclosed T-shaped maze with transparent walls. We will describe in detail the design of our apparatus, the protocol and expected results in healthy wild-type mice.

## Before starting

Organize animal housing (as previously described in^57^) and setup the experimental equipment before starting the procedure.

### Animals

1. After purchasing mice from an external commercial source or acquiring them from an internal mouse breeding facility, transfer them to the behavioural testing facility.
2. House mice in transparent plastic cages with at least 33 cm of length and 15 cm of width, in order to ensure sufficient space for horizontal movement. The height of the cage should be 13 cm, in order to allow complete vertical rearing. According to EU regulations (Directive 2010/63/EU), minimal enclosure size must be 330 cm^2^ (with a minimal floor area per mouse ranging from 60 cm^2^ for mice under 20 g to 100 cm^2^ for mice over 30 g), while minimal height must be 12 cm. As top lid of the cage, use a metal wire grid, which enables climbing and wire-gnawing behaviour, fundamental for mice that are endowed with continuously growing teeth. Provide each cage with an atoxic sawdust bedding. For the bedding, aspen wood should be utilized, while pine and cedar woods, rich in aromatic phenols, should be avoided due to their respiratory and epatic toxicity^58–60^. Furthermore, free choice experiments have shown that mice allowed to choose their nesting material display a natural preference for aspen wood and a strong aversion for pine and cedar woods^60^.
3. House mice socially in order to avoid social isolation stress, which can cause increased anxiety and neophobia, enhanced aggressiveness, locomotor hyperactivity, impairment of short-term and long-term memory, alteration of sensorimotor gating and loss of cognitive flexibility^61–66^. Number of mice should not exceed 4–5 per cage, in order to avoid overcrowding stress, which can lead to increase of corticosteroids and anxiety^67^. When housed socially, mice tend to form social hierarchies and, especially in male mice, this can lead to territorial behaviour and fights for territory^68^. An international data crowdsourcing project collecting records from a sample population of 137,580 male mice distributed across 44 animal facilities revealed a mean facility-level prevalence of aggression-related injuries equivalent to 15 in 1000 mice^69^. For some strains with a high propensity to fight for territory, as Swiss mice^70^, individual caging of male mice could be considered to prevent physical damage and stress from repeated intra-group fights^68,71^.
4. Environment of the housing room should be kept in conditions of controlled temperature (21.5 ± 1 °C) and humidity (40 ± 10%). Through a light timer, program a fixed light/dark cycle of 12 h:12 h, in order to guarantee constant circadian rhythms. If it is possible to obtain animals that were born and raised with inverted light cycles (lights off in the morning), this option should be preferred and animals should be kept under a program with lights off in the morning also in the behavioural facility, so that mice could be tested in the dark phase, which is their active phase^72^.
5. Environmental enrichment of cages, when compatible with the experimental design, should be considered to maximize animal welfare^73–77^. Basic enrichment can feature paper tissue for nesting material and a small plastic nest box into which mice can find shelter. On the other hand, for a more complex environmental enrichment these elements can be combined with tunnels, ladders, hammocks, see-saws, toys or even running wheels.
6. Change home cages once a week, in order to ensure a clean environment. Do not set a higher frequency since cage change is a source of stress for mice, due to removal of odour marks and nesting arrangements. Cages should never be changed on the day preceding a behavioural procedure. Ensure at least 48 h of habituation to the new home cages prior to behavioural testing.
7. Mark each mouse in a way that makes it individually identifiable. Rodents can be marked through several different methods, which include acting on ears, coat, tail or paws. The most commonly employed methods are ear notching, ear tags, tail marking, toe clipping, dye spots on the coat, skin tattooing and implantation of subcutaneous transponders^78^. These marking methods do not share the same degree of stress for the animals. Implantation of transponders, which requires a surgical procedure, is the most invasive method. Tattooing requires puncture of the skin to introduce the ink into the skin tissue. Toe clipping implies removal of a digit, leaving the mouse with a permanent damage to the paw, which could affect ability to interact with objects, grip strength and swimming. This method should always be avoided if alternatives are possible. It should be used only if there are specific reasons related to the experimental protocol and it requires a specific approval from the institutional ethics committee and the national competent authority. In any case, its usage should be limited to newborn mice (up to 10 days of age). Older mice must be clipped under anaesthesia. Ear tagging has the inconvenient that tags can be pulled away by mice during grooming or fighting. Ear notching may be preferable compared with these methods, but it still requires to make small holes on the ear with a special punching tool. The only totally non-invasive marking methods are tail marking and usage of dye spots on the fur. However, with these methods marking must be repeated regularly, as the colour slowly disappears, also due to self-grooming and allo-grooming behaviours.
8. Provide food and water ad libitum (unless the specific experimental design requires animals to be kept on a food-restricted and/or water-restricted regimen).
9. For both mice that have been purchased from an external commercial source and for mice that have been transferred from an internal breeding facility that is separated from the behavioural testing facility, provide at least 1 week for acclimatization to the new environment and recovery from the transportation stress. Indeed, transportation stress can lead to significant alterations of immune, endocrine, cardiovascular, nervous and reproductive systems, as well as of behaviour^79–86^.

### Apparatus

**Figure 1:**
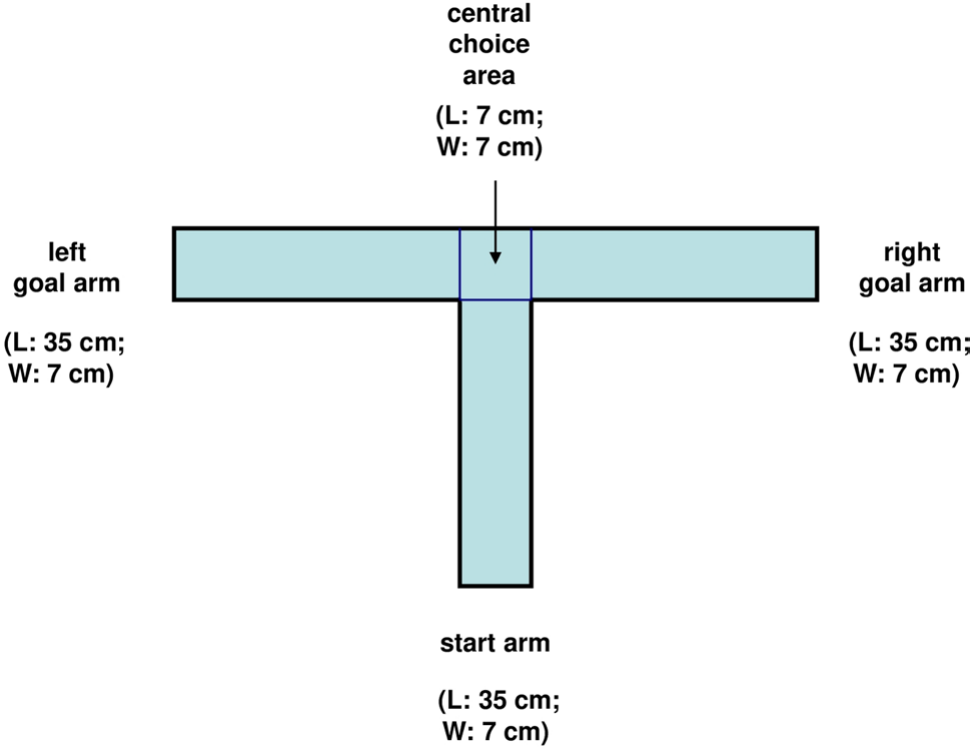
Schematic of the T-maze apparatus. Each arm (start, left and right) is 35 cm long. Central choice area is a 7 cm × 7 cm square. Total width of the maze (from distal end of the left arm to distal end of the right arm) is 77 cm. Total length of the maze (from distal end of the start arm to northern wall) is 42 cm. The blue lines indicate the positions of the three vertically sliding doors.

Purchase a commercial T-maze or build a customized version. In the present paper, spatial working memory was assessed by measurement of spontaneous alternation in a polymethylmethacrylate (PMMA) T-maze (designed by: Raffaele d’Isa; assembled by: Artigianplast, Milan, Italy), mounted 60 cm above the floor. A schematic of the apparatus is represented in Fig. 1. All arms (start arm and lateral goal arms) are 35 cm long and 7 cm wide. The central choice zone is a square 7 cm × 7 cm area. Total width of the maze (from end of left arm to end of right arm) is 77 cm. Total length of the maze (from southern edge of the start arm to northern wall) is 42 cm. The floor of the maze is made of opaque grey PMMA. Transparent lateral walls made of PMMA and 15 cm high prevent falls from the maze, while allowing the vision of extra-maze cues. PMMA thickness is 1 cm for the floor of the maze and 5 mm for the lateral walls. Each arm is endowed with a guillotine door made of transparent PMMA. Doors are 16 cm high, 7 cm wide and 2 mm thick. Considering the central choice area, the three doors are positioned on the southern side of the square (for the start arm), on the western side of the square (for the left goal arm) and on the eastern side of the square (for the right goal arm). The southern door prevents animals that stop in the central choice area from moving back to the initial start arm. On the other hand, the western and eastern doors are used to confine the animals in the goal arms after a choice has been made. The vertically sliding doors are held in place by lateral guides made of transparent PMMA and are completely removable. The four angles of the rectangular guillotine doors are smoothed in order to allow better sliding and removal. At the beginning of each trial, all doors are extracted from the maze and no arm is blocked. Concerning videorecording, the grey colour of the floor allows to distinguish from the background both light- and dark-furred mice, enabling path tracking through video-tracking systems. The floor is opaque, not lucid, in order to avoid strong light reflections. No metallic part has been employed for the assembly of the maze, which can be used also in areas with static and dynamic magnetic fields. Regarding disinfection, the maze has excellent resistance to ultraviolet (UV) light, good resistance to slight contact with ethanol 30% and is not affected by hydrogen peroxide up to 40 volumes (hydrogen peroxide 12%). Highly concentrated solutions of alcohol or hydrogen peroxide can cause cracking and their use should hence be avoided. Autoclavation (which employs temperatures over 120 °C) should not be used as method of sterilization. Temperature resistance of the T-maze apparatus is from − 40 to + 65 °C.

## Materials and equipment

- T-maze apparatus (see previous section for description and measurements)
- Data sheet for behavioural evaluation, containing blank spaces for general information (date, code of the experiment, name of the experimenter) and experimental variables (identification code of the mouse, chosen arm in trials from T0 to T6, choice latency in trials from T0 to T6, total number of correct alternations)
- Pen
- Stop-watch
- Ethanol 30%
- Tissue paper

## Step-by-step procedure

1. Select a quiet and dimly lit room, different from the housing room, as experimental room for the behavioural testing.
2. Locate the T-maze in the desired position inside the testing room. Always keep this position for all trials and all mice. If multiple sessions are performed in different days, the maze must be placed in the same position of the first session. Extra-maze visual cues must remain constant throughout testing.
3. Check functionality of the timer: start, stop and reset functions.
4. Randomize order of mouse testing by experimental group and within-cage order. It has been shown that within-cage order of testing can affect both endocrinological stress responses and behaviour. Corticosterone levels of behaviourally non-tested C57BL/6 mice were significantly elevated after behavioural testing of a cage mate^68^. Moreover, anxiety responses in elevated plus maze significantly differed between first and second tested mice^87^.
5. In the sample trial (T0), place the mouse at the distal end of the start arm, with its head oriented towards the southern end of the maze. Tail-picking should be avoided. Non-aversive handling techniques as open hand handling and tunnel handling should be employed (see the “Animal handling techniques to retrieve the mouse from home cage and from T-maze” section).
6. Let the mouse free to explore.
7. When the mouse turns its head towards the central zone, start the timer.
8. When the mouse enters a goal arm with all four paws and all the tail (tail tip criterion), close the guillotine door behind it and note down chosen arm (L for left or R for right) along with the latency to choose.
9. Confine the mouse in the chosen arm for 30 s. At the end of the confinement period, gently remove the animal and place it back to the distal end of the start arm, stopping and resetting the timer.
10. For the 5 test trials T1–T5, repeat points 5 to 9 for 5 times.
11. For the last test trial (T6), repeat points 5 to 8.
12. Remove the mouse from the T-maze and return it to its home cage.
13. Clean the maze. At first, by using paper tissue, dry urine and pick up fecal boli if present. Subsequently, use paper tissue soaked with ethanol 30% to disinfect arms, walls and doors. This is important to remove intra-maze visual and odour cues that otherwise would not be equal across trials and mice.
14. Wait until the maze is completely dry (the smell of ethanol can be irritating for mice).
15. Repeat points 5 to 14 for all experimental mice.
16. Bring the home cages of the mice back to the housing room.
17. Check and clean from urine and faeces the floor of the experimental room.
18. Clean and put away all equipment.

### Note

Fix maximum time allowed to complete a trial. For test trials, maximum time limit should be 180 s, since with a delay over 3 min working memory becomes too weak. A time limit should be fixed also for the sample trial, in order to control for anxiety-related or motivational factors. Healthy mice generally perform the sample trial in less than 15–20 s. Cut-off time for the sample trial should be 60 s. If, on any trial, an animal exceeds the time limit, gently remove it from the maze and place it back into its home cage. Invalidate the test and retest the mouse after at least 30 min. Restart from T0. Only a sequence of 7 consecutive trials (1 sample trial plus 6 test trials) can be considered a valid test.

### Animal handling techniques to retrieve the mouse from home cage and from T-maze

Lifting by the tail is a commonly used method of handling mice in laboratories^88^. Nevertheless, tail lifting, tail suspension and swinging over the void can be highly stressful for mice. Non-aversive handling methods, as open hand retrieval, are preferable to minimize handling stress and should always be considered. Mice picked by the tail, compared to mice retrieved with open hands, showed increased anxiety in elevated plus maze^89^ and in open field test^90^, higher aversion for the handler in a voluntary interaction test^89^ and reduced exploratory behaviour^91^. We report here a detailed description of an open hand retrieval technique to recoup mice from the home cage and from the T-maze.

Retrieval from home cage:

1. Gently slide the open hands under the mouse moving laterally from the animal’s sides. Unite the hands and curve them as to form a cup hosting the mouse (cupping technique).
2. Only when the mouse has all four paws on the hands, lift it. Bring one hand on top of the other, making it a curve lid for the cup hand hosting the mouse (nutshell hold).
3. Bring the mouse to the start arm of the T-maze. Position the hands over the initial part of the arm and remove the lid hand. Extend the fingers of the cup hand in order to make the hand plain and lean the hand, inclined by approximately 45°, on the maze, with the fingers ending on the arm. Let the mouse spontaneously walk down the hand.

Retrieval from T-maze:

1. Lay the back of the hands on the arm containing the mouse (one hand frontally and one behind the mouse). Gently slide the hands under the mouse. Unite the hands and curve them as to form a cup hosting the mouse.
2. Only when the mouse has all four paws on the hands, lift it. Bring one hand on top of the other, making it a curve lid for the cup hand hosting the mouse.
3. Bring the mouse to the target destination. If the mouse needs to be released in the start arm, position the hands over the initial part of the arm and remove the lid hand. Extend the fingers of the cup hand in order to make the hand plain and lean the hand, inclined by approximately 45°, on the maze, with the fingers ending on the arm. Let the mouse spontaneously walk down the hand. If the animal has concluded the test and needs instead to return to its home cage, then perform the same steps as described above, but bring the hands over the cage as target destination. Then remove the lid hand. Extend the fingers of the cup hand in order to make the hand plain and lean the hand into the cage, inclined by approximately 45° with the fingers ending in the sawdust bedding. Let the mouse spontaneously walk down the hand.

Alternatively to open hand retrieval, recouping through a small plastic tunnel also can be used as non-aversive retrieval method^89–93^. The mouse is encouraged to enter the tunnel with one hand gently approaching it from behind. When the mouse is completely inside, the tunnel is lifted and brought to the target destination.

Additional information and useful videos showing non-aversive mouse handling (including both retrieval through the cupping method and tunnel handling) can be found on the website of the UK National Centre for the Replacement, Refinement & Reduction of Animals in Research (https://www.nc3rs.org.uk/video-clips; https://www.nc3rs.org.uk/news/changing-mouse-handling-practice-university-establishment#videos).

### Data analysis

1. For each animal, from T1 to T6, rate visiting the same arm explored in the previous trial as a perseveration (error) and choosing a different arm as a correct alternation.
2. Calculate the percentage of alternation as an index of working memory, by using the following formula: (total number of correct alternations/6) * 100.
3. For each experimental group, verify if time effects are present. Although latencies generally increase over trials, not necessarily a significant increase will be found in the first session, depending also on the state of stress and anxiety of the animals. If a time effect on latencies is observed, it should be present in all experimental groups. If, on the other hand, the effect is present only in one group and not in the control group, there might be a bias due to motor impairment or anxiety/motivational factors. In that case, care must be taken not to interpret the effects of the experimental manipulation solely as effects on memory processes.
4. Compare percentage of alternation of each experimental group against chance level (50%).
5. Compare percentages of alternation and choice latencies between experimental groups.

### Ethics approval and consent to participate

No human subjects participated to the present study.

### Statement on the welfare of animals

All applicable international, national and institutional ethical guidelines for the care and use of animals were followed. All procedures performed were in accordance with the European Union guidelines (Directive 2010/63/EU) and the Guide for the Care and Use of Laboratory Animals of the U.S. National Institutes of Health (NIH) and were previously approved by the San Raffaele Institutional Animal Care and Use Committee (IACUC).

## Expected results

After building a new T-maze apparatus and/or setting up for the first time the experimental environment, it is useful to first test the T-maze with healthy mice. In particular, three key points must be verified: (1) mice should not display side preference (presence of a strong preference for the left or right side suggests that the experimental setting has not been adequately prepared); (2) mice should spontaneously alternate above chance level (generally between 70 and 80%); (3) choice latencies should not be too high: group average latency for the sample trial (T0) should be < 30 s, while group average of the mean test trial latency (T1–T6 mean: mean of the six test trial latencies) should be < 60 s. If one or more of these points fails to be verified, check the “Troubleshooting” section.

**Figure 2:**
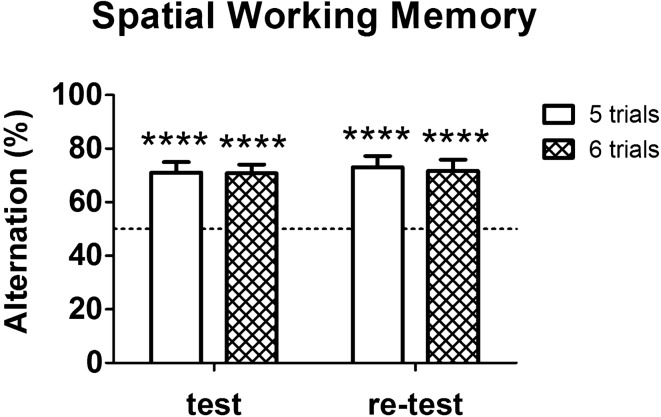
Spatial working memory of healthy wild-type mice in spontaneous alternation T-maze. Percentage of alternation within the first 5 trials (empty bar) and within the first 6 trials (striped bar) were significantly above chance level in both the test and the re-test (for all: *p* < 0.0001). Asterisks highlight significant differences versus chance level (****p < 0.0001). Dashed line represents the 50% chance level. Data are presented as mean + SEM (n = 20).

In order to provide expected outcomes, we tested 20 adult wild-type C57BL/6 male mice (purchased from Charles River, Calco, Lecco, Italy) using the apparatus and the procedure described above. Mice were tested in one session and re-tested 1 week later. Spatial working memory is shown in Fig. 2. Percentages of alternation within the first 6 test trials were significantly above chance level (50%) in both the test (One-sample t-test: t_19_ = 5.294, *p* < 0.0001) and the re-test (One-sample t-test: t_19_ = 5.151, *p* < 0.0001). For mice with particular health conditions or motor limitations it could be useful to use a shorter protocol: 5 test trials instead of the standard 6 test trials. Hence, in Fig. 2, we additionally report percentages of alternation within the first 5 trials. Also in this case, percentages were significantly above chance level in both the test (One-sample t-test: t_19_ = 5.294, *p* < 0.0001) and the re-test (One-sample t-test: t_19_ = 5.510, *p* < 0.0001). No difference was observed between the test and the re-test, for both the 6-trial alternation percentage (Paired samples t-test: t_19_ = 0.165, *p* = 0.871) and the 5-trial alternation percentage (Paired samples t-test: t_19_ = 0.357, *p* = 0.725).

**Figure 3:**
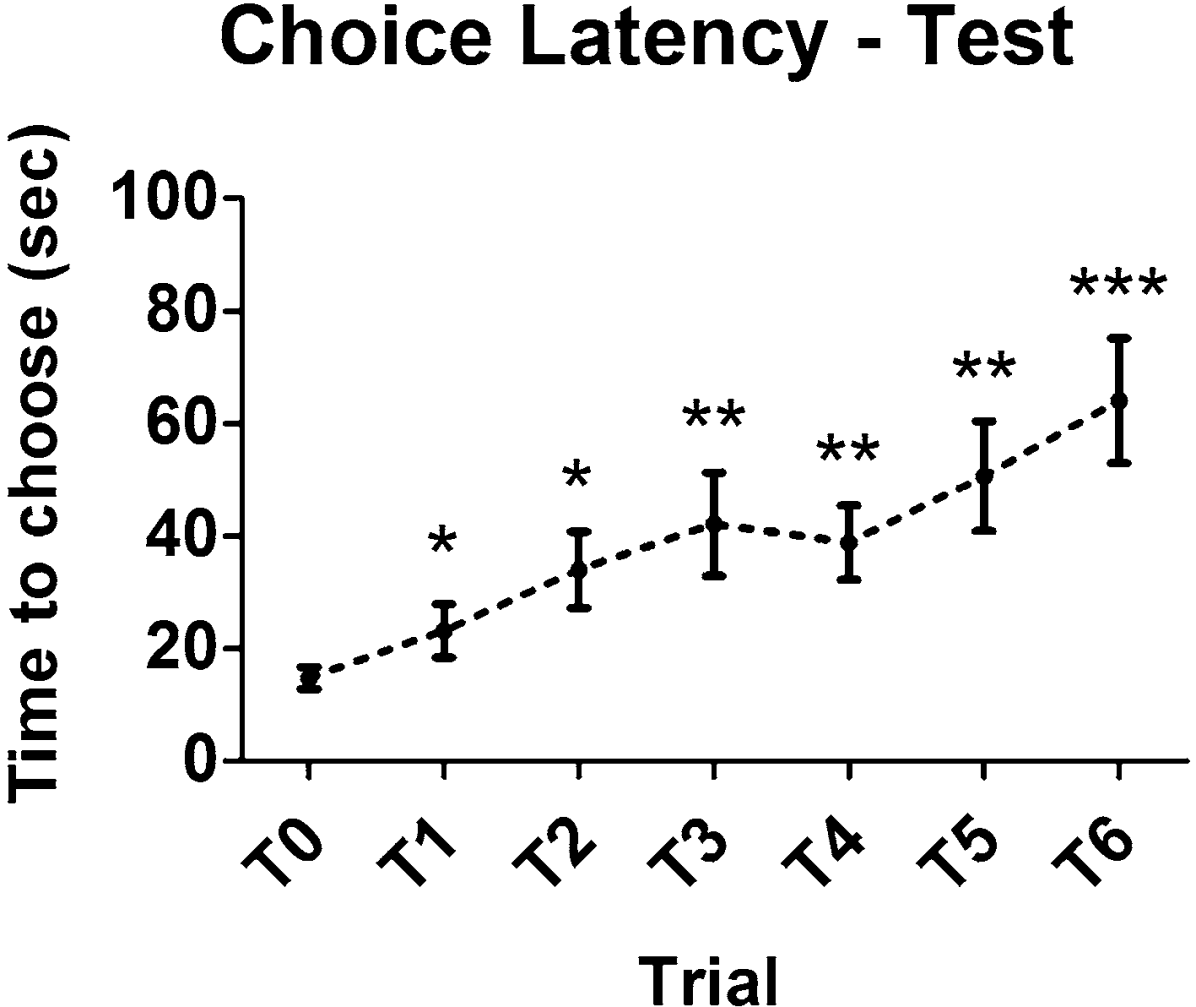
Choice latency of healthy wild-type mice in the test session of spontaneous alternation T-maze (intra-session effect of repetition). A significant increase of choice latency across trials of the test session was found (F_6,114_ = 6.234, *p* = 0.0009). Asterisks highlight significant differences versus sample trial latency (*p < 0.05, **p < 0.01, ***p < 0.001). Data are presented as mean ± SEM (n = 20).

**Figure 4:**
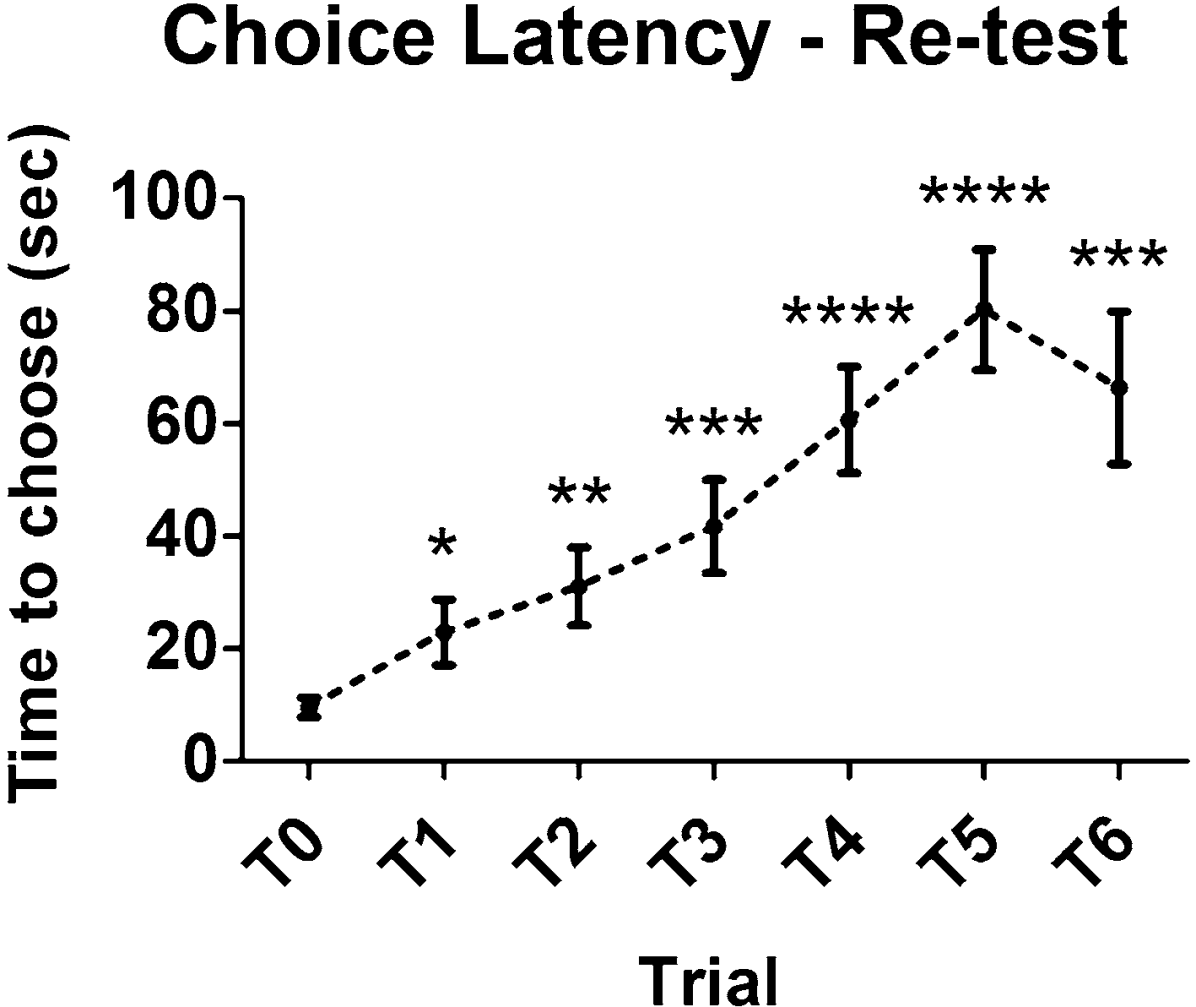
Choice latency of healthy wild-type mice in the re-test session of spontaneous alternation T-maze (intra-session effect of repetition). A significant increase of choice latency across trials of the re-test session was found (F_6,114_ = 10.821, *p* < 0.0001). Asterisks highlight significant differences versus sample trial latency (*p < 0.05, **p < 0.01, ***p < 0.001, ****p < 0.0001). Data are presented as mean ± SEM (n = 20).

**Figure 5:**
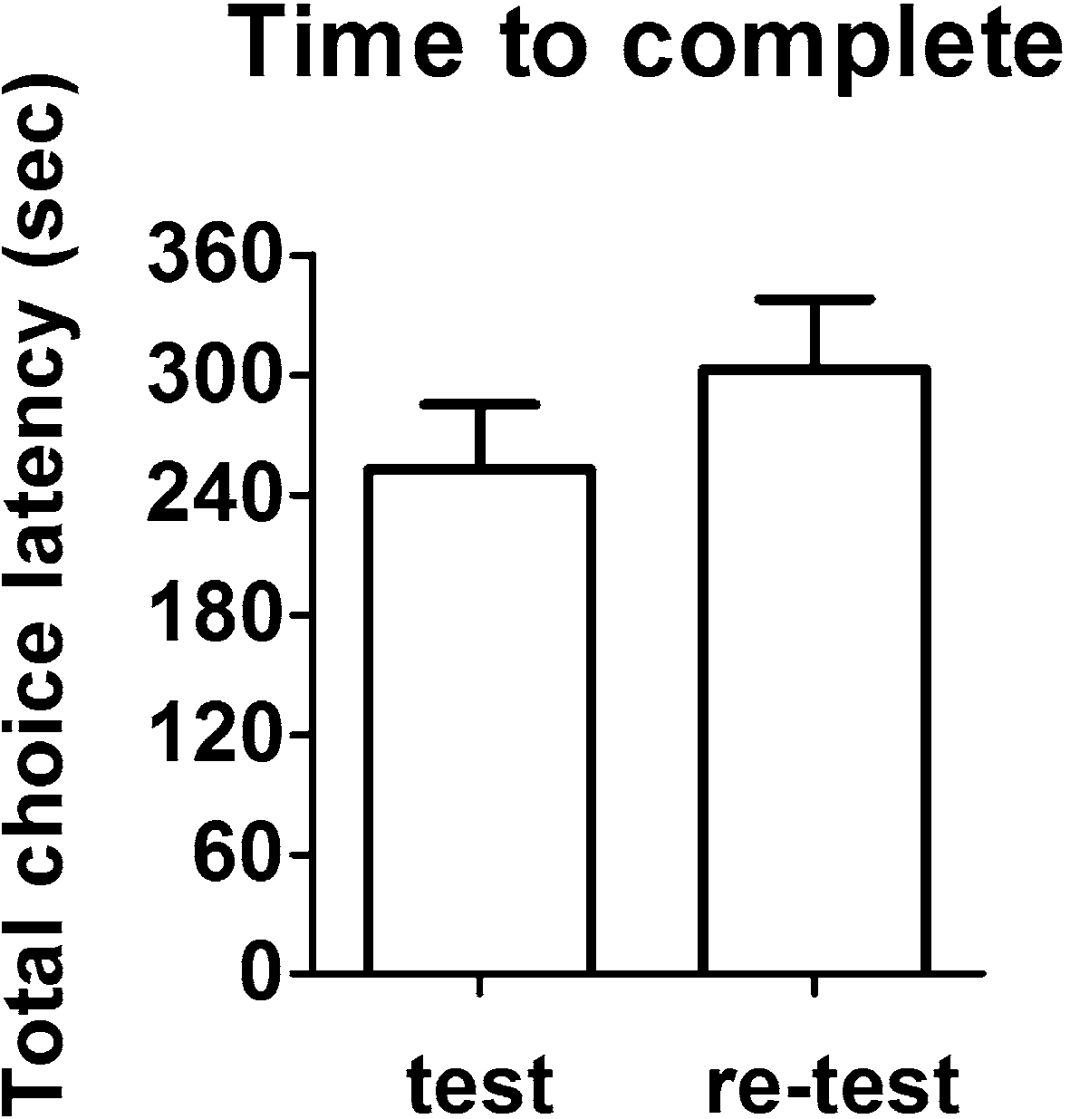
Total choice latency (from T0 to T6) of healthy wild-type mice in the test and re-test sessions of spontaneous alternation T-maze (inter-session effect of repetition). No significant difference was observed between total choice latencies of test and re-test sessions. Data are presented as mean + SEM (n = 20).

Choice latencies for the test session are displayed in Fig. 3. Intra-session effects of repeated exposure to the maze were analyzed. It is common to observe an increase in latencies from T0 to T6. In our results, a repeated measures one-way ANOVA with trial as repeated factor revealed a significant increase of latency over trials (F_6,114_ = 6.234, *p* = 0.0009). Compared with the sample trial choice latency, all test trial latencies were significantly higher (LSD post hoc, T1: *p* = 0.042; T2: *p* = 0.013; T3: *p* = 0.005; T4: *p* = 0.001; T5: *p* = 0.002; T6: *p* = 0.0003). Choice latencies for the re-test session are shown in Fig. 4. Analogously to the test session, also in the re-test a significant intra-session effect on time to choose was observed. Choice latencies significantly augmented over trials (One-way ANOVA for repeated measures: F_6,114_ = 10.821, *p* < 0.0001). Compared with the sample trial choice latency, all test trial latencies were significantly more elevated (LSD post hoc, T1: *p* = 0.013; T2: *p* = 0.003; T3: *p* = 0.0006; T4: *p* < 0.0001; T5: *p* < 0.0001; T6: *p* = 0.0006). Repetition of T-maze testing sessions generally leads to an increase of choice latencies, which in certain cases can be significant already from the first repetition, also depending on the condition of stress of the animals. Inter-session effect of repetition is shown in Fig. 5. Comparison between total choice latency (sum of choice latencies from T0 to T6) of test and re-test is presented. In our results, a 20% increase in total choice latency was found, though this rise was not significant (Paired samples t-test: t_19_ = 1.427, *p* = 0.170).

## Advantages and limitations

Spontaneous alternation T-maze has numerous advantages over other tests of cognitive function. First of all, being based on a natural behaviour of rodents it does not require experimenter-imposed sources of motivation, like rewards or punishments. Animals do not need to be food-deprived or water-deprived to be motivated to find, respectively, a food or water reward. No painful stimuli, such as electrical footshocks, are employed. No immersion in high water is needed to induce movement, as in the Morris water maze or the forced swimming test, which both rely on a highly aversive source of motivation. For these reasons, spontaneous alternation T-maze is considerably less stressful than tests that are not based on a spontaneous behaviour. Along with the object recognition test^94^, it is among the most animal-friendly options for cognitive function assessment. Stress minimization during behavioural procedures is not only fundamental from an ethical point of view for animal welfare, but also for data reliability, as stress is a confounding variable that can put at risk the reproducibility of results^95,96^. Furthermore, the spontaneous alternation T-maze task does not require any sort of pre-training, making it possible to test animals through a single-day procedure, which can be useful in many experimental designs.

However, since the test is based on a spontaneous behaviour, a limitation is that the procedure depends on the state of motivation of the animals. Mice with scarce interest for exploration, as for example mice that have been subjected to chronic stress, with the intention to induce depression-like behaviours, could be more difficult to test in this procedure.

Additionally, mice with severe motor impairments could also be unable to execute this task. Since working memory rapidly fades away within few minutes after removal from the chosen arm, it is important that mice complete trials within the fixed cut-off time. For instance, mice systematically completing trials in more than 5 min due to motor limitations would not be able to be tested with this procedure. Abolition of the cut-off is not a recommended option, since an apparent cognitive defect would actually be secondary to the motor impairment. This can be demonstrated by observing that by releasing the motor-impaired mice at the central end of the start arm, choice latencies no longer exceed the time limit and the cognitive defect disappears. In various cases motor limitations can be overcome by adapting the protocol and establishing a new releasing point: immediately before the square choice area, instead of the distal end of the start arm (the same releasing point must be used for all groups, including the control group). Nevertheless, in some cases choice latencies will still be over the cut-off limit and mice will need to be excluded from the experiment.

## Troubleshooting

• Problem 1: Mice get progressively slower across trials and at a certain point they stop exploring the maze.

Potential solution: mice physiologically increase choice latencies across trials and across sessions. Normally a session of 7 trials (1 sample plus 6 test trials) can be repeated up to three times. Over 3 repetitions of the test, an increasing number of mice will start to exceed the time-out limit, often irrecoverably, and will have to be excluded from the experiment. When deciding which experimental design will be applied, it is better to program no more than 3 sessions of T-maze. If the experimental design requires 4 or more sessions, a higher sample size should be considered in order to compensate for potential loss of experimental subjects (an increase of 40–50% with respect to the desired final sample size should be used). Nevertheless, in certain cases some mice could display high latencies also in the first three sessions. In this case, auditory or tactile stimulations can be used to facilitate movement of mice along the maze. It is important to underline that if this option is chosen, well-defined criteria must be adopted and all mice of all groups must be tested applying the same criteria. Suggested criteria for immobility management are: at 80 s, use acoustic stimulation (snap fingers three times from ~ 30 cm above the centre of the mouse’s body; do not snap from the left or right side, otherwise spatial biases will be induced); at 100 s, gently caress the mouse three times on the centre of its back with the cotton end of a long cotton swab (length: 15 cm; diameter: 0.5 cm; Gima s.p.a., Gessate, Milan, Italy); at 120, 140 and 160 s, repeat tactile stimulation as described above. If the animal exceeds the time-out limit despite acoustic and tactile stimulation, place it back to its home cage and test it again at least 30 min later. Care must be taken to perform the stimulations adequately and avoid that they could be perceived as stressful by the mice. Tactile stimulation should be just a very light touch on the animal’s fur, without pricking, pushing or hurting it in any way. On the other hand, auditory stimulation should be performed by producing a soft and not excessively loud sound.

Some researchers prefer not to use additional auditory or tactile cues. Three options are possible: (1) no use of auditory/tactile stimulation; (2) use only on mice that went time-out; (3) default use on all mice. Our suggestion is to adopt the additional stimulation only when it is strictly necessary, that is in experiments with a high number of time-outs and exclusively on mice that previously went time-out. In any case, when one of the three rules has been chosen, it must be kept constant across all the experiment. The representative results from healthy wild-type mice reported in the present work have been obtained without the employment of any auditory or tactile stimulation (on 280 trials no time-out was observed).

• Problem 2: Mice keep coming back into the start arm instead of choosing a goal arm.

Potential solution: When a mouse stops in the central choice area, close the door of the start arm behind it, in order to prevent it from returning to the initial arm.

• Problem 3: Mice show continuous rearing on hindlimbs and sniffing of the air instead of moving along the maze.

Potential solution: Check if olfactory stimuli in the experimental room are distracting the animals (for instance, if a previous operator has performed blood sample collection and has not cleaned well). Remove the olfactory distraction.

• Problem 4: Mice lick the maze instead of exploring.

Potential solution: Check if a fruit-fragranced washing solution has been used to clean the maze. If it has, use paper tissue wet with water to remove the solution from the maze and subsequently use paper tissue soaked with ethanol 30% to eliminate any residual scent.

• Problem 5: Mice release abundant urine and faeces while in the maze.

Potential solution: Increase of urination and defecation are signs of anxiety. Something in the room might be disturbing the animals. Check that the experimental room actually was set to be in conditions of dim light and not accidentally left in standard illumination (since mice are naturally photophobic, bright lights can generate anxiety) and that there are no sources of loud noise which can scare rodents.

• Problem 6: Mice run too fast and it is difficult to note down in time all the outcomes.

Potential solution: When a mouse chooses a goal arm, look at the stopwatch and keep in mind the latency. After closing the door of the chosen goal arm, exploit the confinement period to write down chosen side and latency of choice. A pre-printed sheet with the variables to fill in for each trial is also useful to optimize time during the behavioural test. Alternatively, observation programs as Behavioral Observation Research Interactive Software (BORIS) can be used.

• Problem 7: Control mice show chance level alternation.

Potential solution: This is uncommon in healthy adult mice. Make sure that there are not sources of auditory, visual or olfactory distraction in the experimental room during the test. Verify that housing conditions are regular and not stressful. Check that home cage has not been changed on the day preceding the test or on the day of the test and that mice have not undergone stressful manipulations in the previous days (as anaesthesia and surgery, after which at least 5 days of recovery must always be granted before behavioural testing).

• Problem 8: Control mice show strong lateralization of choices.

Potential solution: If healthy mice show a strong tendency to turn left (or right) there could be a source of attraction on one side or of aversion on the other. For example, if male mice are being tested and on a desk at the left of the maze there is an open cage with female mice, a strong tendency to turn left can be observed. On the other hand, if lighting is not uniform on the maze and one arm is more brightly lit than the other, this arm could be perceived as aversive and hence avoided.

• Problem 9: Motor-impaired mice move slowly along the start arm and arrive to the goal arm over the cut-off time limit.

Potential solution: Establish a new releasing point. At the beginning of each trial place mice at the central end of the start arm (just before the square central choice area) instead of at the distal end. The same releasing point must be used for all experimental groups, including control mice.

• Problem 10: After being left on the maze, mice do not turn around.

Potential solution: This indicates that a mouse may be ill, lethargic or very old. Gently help manually the mouse to turn around. If the mouse starts then to move along the start arm, let it explore freely the maze. If the mouse stays immobile, adopt a shaping strategy and gently guide it manually along the start arm and along one of the two goal arms. Repeat this familiarization procedure until the mouse turns spontaneously and moves along the maze, up to a maximum of 3 times. If the mouse explores autonomously, then start the testing session (from T0). If the animal is still immobile, take it out from the maze and place it back into its home cage. Retest the mouse few hours later.

• Problem 11: Mice tend to jump off the maze.

Potential solution: This is uncommon, but it could happen in some cases with juvenile mice or with genetic mutations, pharmacological manipulations and brain lesions leading to hyperactivity. Cover the three arms with transparent film, leaving open only the central choice arena, in order to allow closure of the guillotine doors. When retrieving mice at the end of a trial, temporarily lift the film and cover again arms after the retrieval. Alternatively, increase the height of the side walls of the maze (50 cm should be enough to prevent escapes through jumps).

## Data Availability

Data and further information on the T-maze apparatus are available upon reasonable request.

## Code availability

No new software application or custom code was developed for the present paper.
